# Supplementary material for: Simultaneous pancreas and kidney transplantation: A qualitative study of partners’ experiences
Source: PLoS One. 2024 Nov 18;19(11):e0313907. doi: 10.1371/journal.pone.0313907 (PMC11573160; doi:10.1371/journal.pone.0313907)
Supplement: S1 File — (DOCX) [file pone.0313907.s001.docx]

**Simultaneous pancreas and kidney transplantation: A Qualitative study of partners’ experiences - Interview topic guide**

**Questions**

1. **First, could you please tell me about your experience before your partner received a pancreas and kidney transplant?**

- What was your experience when your partner just had diabetes, before the kidney problems?
- Did you need to support your partner with their diabetes and in what sort of ways?
- When and how did you discover that your partner was having kidney problems?
- How did the kidney disease develop?
- Did you need to support your partner because of the kidney disease and in what ways?
- How well did you feel that you understood your partner’s health conditions and the treatment?

1. **What sort of impact, if any, did your partner’s diabetes and treatment have on you and your quality of life?**
2. **What sort of impact, if any, did your partner’s kidney condition and treatment have on you and your quality of life?**

- Work/social life/leisure activities/role around the house/other family/ holidays/ dietary?
- Did your partner’s diabetes and kidney disease impact on your relationship and in what way?
- What sort of changes, if any, did you need to make due to your partner’s health conditions?

1. **What was the process like when your partner was added to the transplant waiting list?**

- How did you feel about your partner being on the waiting list for a pancreas and kidney transplant?

1. **What sort of strategies did you use to help you cope before the transplant?**
2. **Next, could you please tell me about the time when your partner received their pancreas and kidney transplant?**

- What happened?
- What was the recovery period like?
- How did you feel during this time?

1. **How has the transplant impacted your quality of life?**

- Work/social life/partner relationship/leisure activities/role around the house/other family/holidays?
- What has been most impacted or improved for you?

1. **Are there ways in which your partner’s health conditions are still impacting on your life and in what way?**
2. **In what ways, if any, do you still support your partner now in relation to having had these conditions?**

**Now I would like to discuss with you how you have adjusted and how you cope with your partner** **having a pancreas and kidney transplant.**

1. **How have you adjusted to your partner having the pancreas and kidney transplant?**

- Have there been any changes that you have had to adjust to and in what way?
- Have you experienced any changes in your relationship and in what way?
- Is there anything you think might have helped you both cope throughout the process?
- What sort of resources have you used to help you cope throughout the process, if any?
- Have there been any difficulties or issues since the transplant and how have you coped with them?

1. **When your partner was waiting for the pancreas and kidney transplant what expectations did you have about life for you both after the transplant, if any, and have they been met?**

**The closing**

Thank you…Now we are nearing the end of the interview.

1. **Is there anything else you can think of that would be helpful for me to know to have an accurate record of your experience?**
