# Supplementary material for: Simultaneous pancreas and kidney transplantation: A qualitative study of partners’ experiences
Source: PLoS One. 2024 Nov 18;19(11):e0313907. doi: 10.1371/journal.pone.0313907 (PMC11573160; doi:10.1371/journal.pone.0313907)
Supplement: Attachment — Submitted filename: PONE-D-23-40586 R1 Response to Reviewers.docx [file pone.0313907.s003.docx]

RE: PLOS One PONE-D-23-40586 – R1

Dear Editor

Thank you for the opportunity to revise and resubmit the paper *Simultaneous pancreas and kidney transplantation: A qualitative study of partners’ experiences.*

Please see our responses to the comments from the editorial team and the reviewers below.

*Editorial team comments:*

1. *Please ensure that your manuscript meets PLOS ONE's style requirements, including those for file naming. The PLOS ONE style templates can be found at https://journals.plos.org/plosone/s/file?id=wjVg/PLOSOne_formatting_sample_main_body.pdf and* [*https://journals.plos.org/plosone/s/file?id=ba62/PLOSOne_formatting_sample_title_authors_affiliations.pdf*](https://journals.plos.org/plosone/s/file?id=ba62/PLOSOne_formatting_sample_title_authors_affiliations.pdf)

**We have checked and amended the formatting of the manuscript so that it now complies with the style requirements of PLOS ONE. These amendments have not been highlighted in the tracked change document to allow for greater readability.**

2. *…Please state what role the funders took in the study. Please include this amended Role of Funder statement in your cover letter; we will change the online submission form on your behalf."*

**We have amended the Funding statement to include the information about the role of the funders.**

*3. We note that you have indicated that there are restrictions to data sharing for this study… If there are ethical or legal restrictions on sharing a de-identified data set, please explain them in detail (e.g., data contain potentially identifying or sensitive patient information, data are owned by a third-party organization, etc.) and who has imposed them (e.g., a Research Ethics Committee or Institutional Review Board, etc.). Please also provide contact information for a data access committee, ethics committee, or other institutional body to which data requests may be sent. We will update your Data Availability statement on your behalf to reflect the information you provide.*

**The de-identified interview transcripts have not been made available as they have been collected within a small group of participants who could be identifiable using basic characteristics used in the analysis (e.g. age, gender etc.). The data also contain potentially sensitive patient medical information that may violate participant privacy. Participants did not consent to data being shared outside of the research team, so to do so would breach ethical approval. The ethics committees concerned were the East of England – Cambridge Central Research Ethics Committee (REC reference: 18/EE/0256), or the London - Queen Square Research Ethics Committee (REC reference: 18/LO/0134) but we suggest that it would not be appropriate to provide wider access to these data.**

*4. Please review your reference list to ensure that it is complete and correct. If you have cited papers that have been retracted, please include the rationale for doing so in the manuscript text, or remove these references and replace them with relevant current references. Any changes to the reference list should be mentioned in the rebuttal letter that accompanies your revised manuscript. If you need to cite a retracted article, indicate the article’s retracted status in the References list and also include a citation and full reference for the retraction notice.*

**We have checked the reference list and have not identified any papers that have been retracted. We have included one additional reference to support points made in response to the reviewers’ comments on data saturation. This can be seen in the tracked change version.**

Reviewers' comments:

*Reviewer #1:*

*Thank you for the chance to review this paper. It represents important perspectives of partners in relation to pancreas and kidney transplants. I have written a couple of questions and points of clarification below. Best wishes forward!*

*Introduction*

*The study is introduced with a relevant literature review and clear rationale for the study. The structure and logic of the text is helpful for readers.*

**Thank you for your comments.**

*Methods*

*The research process is clearly described and using the SRQR checklist is helpful. I have a question about recruitment, 15 partners were invited to participate, and 8 decided to do so. How did you decide that the 15 and the 8 were sufficient? Thinking of the four research questions, it seems it would have been a benefit to have a few more participants.*

**We agree that a larger number of participants could have been beneficial. Our sample was limited by practicalities; due to nature of the study, the pool of potential participants was relatively small. For example, we were limited to participants who had a functioning SPKT and who also had a long-term partner. We were also limited on the number of times that we could reach out by phone to potential participants (a maximum of three attempts) and could only interview those who returned the signed consent form.**

**Despite this, we feel that we had sufficient information power, in that the interviews provided a lot of relevant information, and considering the narrow scope of the study, as well as the specific sample being used, that the data we collected was sufficient. Moreover, the themes we found complement those found in previous research, so we feel confident that our findings are representative of the data we collected.**

*The interview guide is extensive and seems very structured. The concept quality of life is central in several questions. In the introduction, quality of life is mentioned once, as is well-being, health and life satisfaction. Perhaps other types of questions and foci would have generated different findings.*

**We agree that other questions would have generated different findings. However, we are mindful that this is true for all research. This study was part of a larger PhD that examined quality of life and well-being in people with renal or SPK transplants. From this, the schedule did have more of a focus on quality of life in the questions. However, we used reflexive thematic analysis without any specific codebook, so that we could explore further what participants shared with us in interviews. This meant that the findings were not tied to just discussion of quality of life. We have added in further explanation of this in the Recruitment and Procedure section.**

*Findings*

*The findings are presented in six themes. The second theme is presented with two sub-themes, one covering two pages and one about ¼ of a page. I am uncertain about the reasoning behind this choice. In my reading it comes across as confusing and makes me wonder about balance in presentation of findings. Do the sub-themes contribute in a significant way?*

*The theme “Got on with it” is also quite short, perhaps it would make sense to merge it with another theme. My general impression of the findings is that they could be condensed and refined further for greater balance.*

**Thank you of your comments. We agree that the themes would benefit from revision, so we have reviewed the themes, and considered what would be the most useful to present. We now present two main themes: (1) navigating life with an ill partner, and (2) relief and the realities of life post-transplant. The first theme, navigating life with an ill partner, has three subthemes: a) living with pervasive worry, b) stress of enhanced responsibilities, and c) the buffering influence of support. We feel this highlights the findings in a more concise way, whilst still representing the data accurately.**

*Discussion*

*The discussion dives straight into highlighting a few of the findings, fear of hypoglycemia, prioritizing their partner before themselves, getting on with it and relief after the transplantation. Please consider signposting this at the start of the discussion; it would be helpful for readers. Perhaps starting with a brief summary of the main findings could be useful. It may also be helpful to consider the main message of the discussion; what should readers take or learn from these findings. Parts read a bit repetitive in comparison to the results.*

**Thank you for this suggestion; we agree that this would be beneficial. We have added in a short summary of the themes at the start of the discussion, which helps to detail the main message of the study. We have also edited the discussion so that it is more concise, removing sections that were repetitive.**

*The study limitations would benefit from considering a wider scope of the research process. At present only selection bias and gender are commented on. For example, trustworthiness and credibility of the findings could be elaborated.*

**We have added in reference to credibility and reflexivity within the discussion of the limitations of this study.**

*Conclusion*

*The conclusion highlights health and well-being of the participants. Quality of life is not mentioned, which is a bit tricky as it is frequently used in the interview guide.*

**We have edited the conclusion so that quality of life is mentioned.**

*Reviewer #2:*

*An important interview study with partners/caregivers of people requiring a SPK transplant. Some suggestions for improvement include:*

*1. This is a small sample size and I believe that needs to be acknowledged in the limitations paragraph.*

**We have included acknowledgment that the sample is smaller than other qualitative studies and expanded upon this point in the limitations.**

*2. Could you please clarify if all the partners where co-habitating with the person with kidney failure, as this may alter their experiences.*

**Yes, all participants were co-habiting with their partners. This has been included to aid clarity.**

*3. Was data saturation reached. I realize you only interviewed the 8/15 who responded but I want o know in the main body of the paper did you reach data saturation?*

**Data saturation is increasingly considered a less relevant criterion for judging the quality of studies that use reflective thematic analysis (Braun & Clarke, 2021). For this reason, we did not measure data saturation, but we did consider information power, as defined by Malterud et al. (2016). Malterud et al. contend that ‘the more information the sample holds, that is relevant for the actual study, the lower amount of participants is needed’ (p. 1753). In the case of this research, a smaller sample is adequate as we had a narrow research focus and were using a specific group of people (the long-term partners of people who have had a successful SPKT). We have added in details about this in the discussion, to address this point.**

*4. Findings - your themes and subthemes could do with another revision. They are very matter of fact and not conveying the true nature of the theme ie Living with worry- this could be improved to highlight the tre nature of the theme by including persistent or pervasive. Alos some of the themes have large bodies of writing that I feel highlight some subthemes that are important - impact on partners work for an example. Pleases revise and deduce whether further subthems could be highlighted.*

**As mentioned in the response to Reviewer 1, we have reviewed and edited our themes so that they are more interpretive, whilst still reflective of the data and participants’ experiences.**

*5 For contextual assistance was this study conducted in city or rural areas as that does impact burden for partners and highlights further issues.*

**Participants were recruited through SPKT recipients who attended large urban transplant centres across the UK. We have not documented whether participants were from a rural or urban area themselves, so cannot comment further on that. This limitation has been added into the discussion, to acknowledge that there may be differences in people’s experiences.**

Braun V, Clarke V. To saturate or not to saturate? Questioning data saturation as a useful concept for thematic analysis and sample-size rationales. Qualitative Research in Sport, Exercise and Health. 2019;13(2):201–216. [doi: 10.1080/2159676X.2019.1704846](doi:%20%2010.1080/2159676X.2019.1704846)

Malterud K, Siersma VD, Guassora AD. Sample size in qualitative interview studies: Guided by information power. Qual Health Res. 2016;26(13):1753-1760. [doi: 10.1177/1049732315617444](https://doi.org/10.1177/1049732315617444)
